# PathFinder: a novel graph transformer model to infer multi-cell intra- and inter-cellular signaling pathways and communications

**DOI:** 10.3389/fncel.2024.1369242

**Published:** 2024-05-23

**Authors:** Jiarui Feng, Haoran Song, Michael Province, Guangfu Li, Philip R. O. Payne, Yixin Chen, Fuhai Li

**Affiliations:** ^1^Institute for Informatics (I2), Washington University School of Medicine, Washington University in St. Louis, St. Louis, MO, United States; ^2^Department of Computer Science and Engineering, Washington University in St. Louis, St. Louis, MO, United States; ^3^Division of Statistical Genomics, Department of Genetics, Washington University in St. Louis, St. Louis, MO, United States; ^4^Department of Surgery, University of Missouri-Columbia, Columbia, MO, United States; ^5^Department of Molecular Microbiology and Immunology, University of Missouri-Columbia, Columbia, MO, United States; ^6^NextGen Precision Health Institute, University of Missouri-Columbia, Columbia, MO, United States; ^7^Department of Pediatrics, Washington University School of Medicine, Washington University in St. Louis, St. Louis, MO, United States

**Keywords:** Alzheimer’s disease, signaling pathways, cell cell signaling communications, microenvironment, graph neural network

## Abstract

Recently, large-scale scRNA-seq datasets have been generated to understand the complex signaling mechanisms within the microenvironment of Alzheimer’s Disease (AD), which are critical for identifying novel therapeutic targets and precision medicine. However, the background signaling networks are highly complex and interactive. It remains challenging to infer the core intra- and inter-multi-cell signaling communication networks using scRNA-seq data. In this study, we introduced a novel graph transformer model, PathFinder, to infer multi-cell intra- and inter-cellular signaling pathways and communications among multi-cell types. Compared with existing models, the novel and unique design of PathFinder is based on the divide-and-conquer strategy. This model divides complex signaling networks into signaling paths, which are then scored and ranked using a novel graph transformer architecture to infer intra- and inter-cell signaling communications. We evaluated the performance of PathFinder using two scRNA-seq data cohorts. The first cohort is an APOE4 genotype-specific AD, and the second is a human cirrhosis cohort. The evaluation confirms the promising potential of using PathFinder as a general signaling network inference model.

## Introduction

Single-cell RNA sequencing data (scRNA-seq) technologies have become popular in recent years because of their ability to profile gene expression and analyze cell composition in the single cell resolution (Kolodziejczyk et al., 2015; Tanay and Regev, 2017; Hwang et al., 2018). On the one hand, by profiling and annotating scRNA-seq data, researchers can analyze differentially expressed genes in each cell population and sub-population to understand which gene is altered in certain conditions. On the other hand, scRNA-seq data also show great potential in discovering intra- and inter-cellular communication. However, there are only limited methods for discovering active signaling pathways or intra-cellular communication using scRNA-seq data. The existing models are mainly based on correlation, regression, and Bayesian analysis (Saint-Antoine and Singh, 2019), and the direct interaction signaling cascades were usually ignored in those methods because only a small set of genes exhibit gene expression changes between different conditions (Feng et al., 2020). For example, CellPhoneDB (Efremova et al., 2020) can model the interactions between ligands from one cell type and receptors from another cell type. However, it cannot model the downstream signaling. CCCExplorer (Choi et al., 2015) can discover both the ligand–receptor interaction and downstream the signaling network by modeling differentially expressed genes. NicheNet (Browaeys et al., 2020) takes a further step by integrating various interaction databases and training a predictive model to assess the interaction potential between the ligand and downstream targets. However, it only applies a statistical model, which cannot generate a clear communication path. CytoTalk (Hu et al., 2021) applies the Steiner tree to discover the de-novo signal transduction network from gene co-expression. However, the discovered signaling is based on co-expression, and the physical interaction cascade is still unknown.

In the past few years, graph neural networks (GNNs) have become famous due to their great performance in node and graph representation as wells as in classification tasks. For instance, GraphSAGE (Hamilton et al., 2017) proposed the first general framework for learning the node representation inductively. GAT (Veličković et al., 2017) incorporates the attention mechanism into GNNs to actively learn how to aggregate all the information in graphs. The DGCNN (Zhang et al., 2018) model proposes sortPooling to efficiently sort nodes and learn graph features for graph classification. GIN (Xu et al., 2018) connects message-passing GNNs with the 1-dimensional Wifelier-Lehman test (1-WL test) on learning graph structure and proposes a new GNN algorithm that is equally powerful as the 1-WL test. More recently, researchers have tried to generalize the transformer architecture (Vaswani et al., 2017) into graph learning fields as it already shows superior power in learning both text and image data. Many studies (Cai and Lam, 2020; Hu et al., 2020; Rong et al., 2020; Zhang et al., 2020; Yang et al., 2021; Ying et al., 2021) have shown great potential in applying the transformer model to the graph data. They either nest GNN architectures in the transformer layer, design specific attention mechanisms, or design novel encoding mechanisms to incorporate the graph structure into the transformer model. However, using GNNs to discover the intra- and inter-cell communication network remains unknown as these networks are typically black-box models and it is hard to interpret their prediction results.

**Figure 1:**
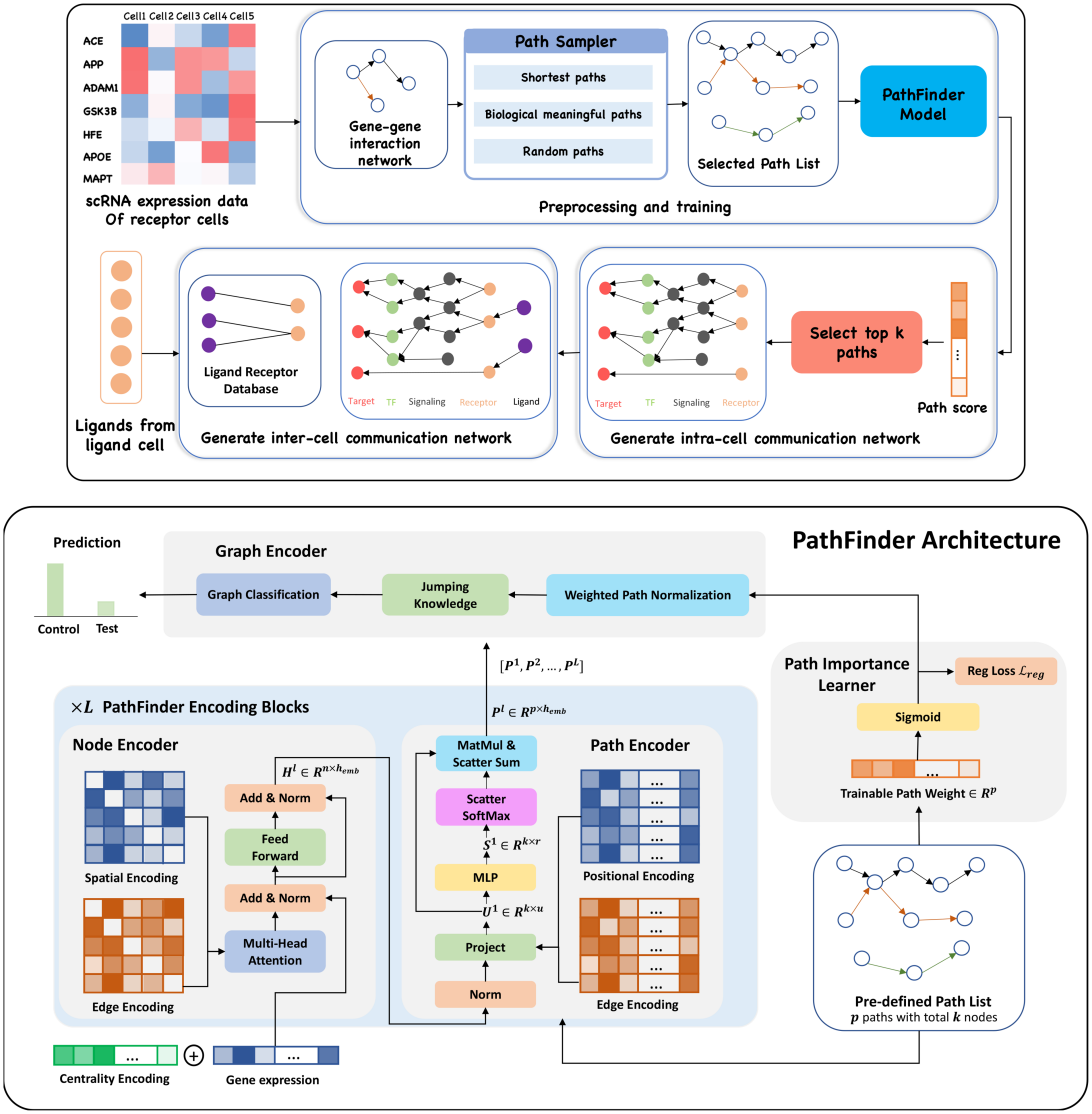
**(Upper)** Overview of the PathFinder method to discover both intra- and inter-cell communication networks. The input scRNA expression data with both samples from the control condition and the test condition are used to construct the gene–gene interaction network based on our large database. Then, the path sampler is used to generate all pre-defined path from the interaction network. Then, the PathFinder model is trained to separate the cells from two different conditions. After the training, the learned path score can indicate the importance of each path. The top k paths are selected to generate the intra-cell communication network. Finally, the ligand–receptor database is used to link all picked ligands (like differential expressed ligands) from ligand cells to the receptors in the intra-cell communication network of receptor cells to construct the inter-cell communication network. **(Lower)** Model architecture of PathFinder. The PathFinder model consists of three components: node encoder, path encoder, and graph encoder. The node encoder is a stack of L the transformer layer with special encoding to encode local graph structure information of each node. The path encoder take the output from each layer of node encoder to learn long-range path embedding for each pre-defined path. Finally, the graph encoder aggregate information from each path to generate graph embedding and make final prediction. In the graph encoder, the trainable path weight will be learned to assign each path an importance score, which can be used to generate intra-cell communication networks.

In this study, we present a novel framework called PathFinder to discover both intra- and inter-cell communication networks with a novel graph transformer-based neural network. Given the scRNA-seq expression data and the condition (control/test), PathFinder first samples a series of predefined paths through the prior gene–gene interaction database. Then, the PathFinder model takes the scRNA-seq expression data and the predefined path list as inputs to predict the condition of each cell. Through the training, the path important score will be learned to indicate the relative importance of each path in separating between the control and test conditions. To learn different types of communication, such as upregulated or downregulated networks, a novel regularization term is introduced. PathFinder will first generate a prior score for each path based on the expression level of genes in the path. Then, during the training, this regularization term will regularize the learned path scores to be close to the prior scores. After training, the path score will be sorted and the intra-communication network for each cell type will be generated by extracting the top K important paths. To generate the inter-cell communication network between the ligand cell and the receptor cell, the intra-cell communication network for the receptor cell will be collected, and the ligand list will be extracted from the differential expressed gene list in the ligand cell. Finally, the ligands are linked to the intra-cell network based on the ligand–receptor interaction database. The overall procedure of generating both intra- and inter-cell communication networks using PathFinder is shown in Figure 1. To thhe best of our knowledge, this is the first method to apply deep learning and graph transformers to discover signaling networks in scRNA-seq data. The advantages of PathFinder are listed below: (1) The model is designed based on a graph transformer, which has the great ability to learn both local and long-range signaling patterns from gene expression and large-scale networks. (2) It is capable of identifying and providing the full signaling network between cells via cellular ligands and receptors. (3) The proposed PathFinder is a general framework that allows users to input their own defined signaling paths or gene–gene interaction network database to identify important signaling based on their interests. Furthermore, (4) it can separate and generate different types of communication networks (Differential expressed/upregulated/downregulated), which allows more precise downstream analysis. We applied the PathFinder model on two scRNA-seq data cohorts: one is a mice cohort of AD and another is a human cohort of cirrhosis. The PathFinder not only achieves great prediction results but also generates intra- and inter-cell communication networks that align well with the latest knowledge on the mechanism of both two diseases.

## Results

### scRNA-seq data of Alzheimer’s disease cohort on mice

To evaluate the proposed PathFinder method, scRNA-seq data on Alzheimer’s disease are collected from the Gene Expression Omnibus (GEO) database with accession number GSE164507 (Wang et al., 2021). The raw data are processed using the Seurat R package (Hao et al., 2021), and the process procedure is conducted by following the previous study’s procedure (Wang et al., 2021). Specifically, we select cell samples from two different conditions, denoted as TAFE4_tam and TAFE4_oil. TAFE4_tam refers to mice with the APOE4 gene knocked out from astrocyte cells, and TAFE4_oil refers to mice with the existence of APOE4. It is well known that APOE4 is one of the most significant genetic risk factors for late-onset AD. By analyzing the difference between the signaling pattern with and without APOE4, we can gain a deeper understanding of the effects of the APOE4 gene on brain cells.

Concretely, the excitatory neuron (Ex), microglia (Mic), and astrocyte (Ast) of the TAFE4 group are collected from the dataset with a total number of samples of 13,604, 3,874, and 734, respectively. The detailed data distribution are provided in Supplementary Table S1. Then, the PathFinder method is applied to predict the condition of each cell (oil or tam) separately for each cell type and generate both intra- and inter-cell communication networks between these three cell types. The pre-defined path list includes all shortest distance paths starting from receptors and all possible paths from the receptor to the target gene. For the shortest distance paths, we only select paths with a minimum length of 3 (except all receptor direct regularizations, which have a length of 2) and a maximum length of 10. We compute the prior score of each path based on the average differential expression level of all genes in the path (more details in the Method section) for the path score regularization. To ensure the robustness of the analysis, we only selected the top 8,192 variable genes from the original dataset as input to the model, which resulted in a final count of 1,210 pre-selected paths. The detailed path selection procedure can be found in the Method section.

### scRNA-seq data of cirrhosis cohort on humans

The scRNA-seq data of human cirrhosis is obtained from the GEO database under the accession number GSE136103, which includes non-parenchymal cells collected from healthy individuals and patients with cirrhosis. After processing, single-cell data were obtained from five healthy individuals (healthy1-5) and five patients with cirrhosis (cirrhotic1-5). Similarly, the raw data are processed using the Seurat R package (Hao et al., 2021). After the process, we select three important cell types: endothelial (Endo), macrophages (Mac), and T cells (Tcell). The total number of cells for each cell type is 6,197, 9,173, and 20,950, respectively. The detailed data distribution is provided in Supplementary Table S1. Similar to the AD cohort, we use PathFinder to predict the cell condition for each cell type. The pre-defined path list is selected in the same way as the AD dataset. For the cirrhosis cohort, we selected the top 12,000 variable genes from the original dataset as input to the model, which resulted in a final count of 1,549 pre-selected paths.

### PathFinder can effectively separate cells from different conditions of AD by selecting differentially expressed signaling paths

**Figure 2:**
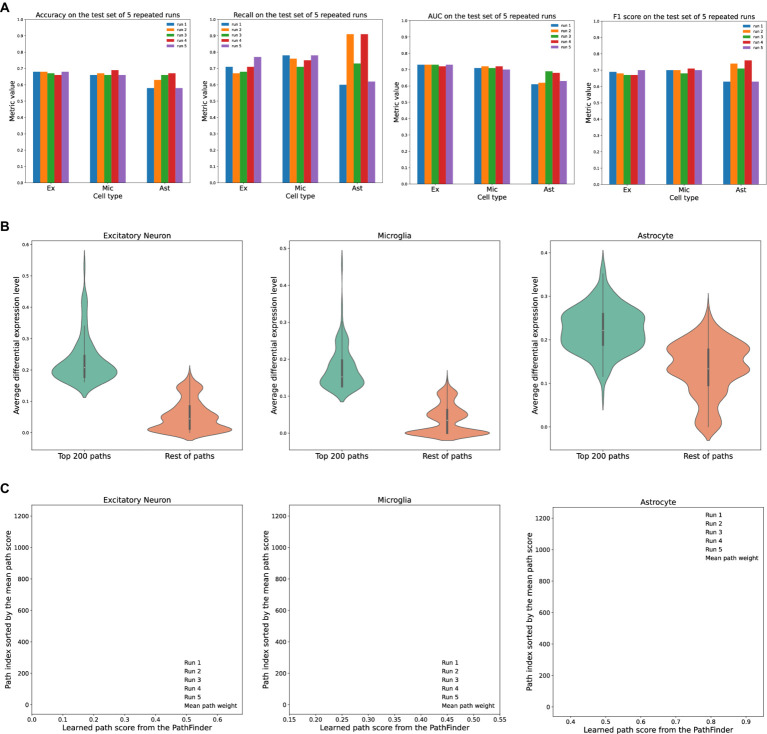
Evaluation of the PathFinder model on the AD cohort. **(A)** The detailed evaluation metrics on test dataset from all runs. **(B)** The comparison of the average differential expression level of top paths sorted by PathFinder during the training. The top 200 paths have higher differential expression level than others for all three cell types. **(C)** The learned path scores of PathFinder on different runs. All paths are ranked by the average score across all runs.

To evaluate the performance of the PathFinder model, it is applied to excitatory neurons, astrocytes, and microglia cells from the AD cohort separately to predict the conditions of each cell (tam/oil), denoted as TAFE4_ex, TAFE4_mic, and TAFE4_ast, respectively. For each cell type, we repeat the training five times, each time randomly splitting the whole dataset into train, validation, and test subsets at a ratio of 0.7/0.1/0.2. We report the average performance and standard deviation on the test set over all five runs. The detailed experimental setting can be found in the Method section. The detailed results are shown in Table 1 and Figure 2A.

**Table 1:** Evaluation results of the PathFinder model.

	Accuracy	Recall	Precision	Specificity	F1	AUC
TAFE4_ex	0.67 ± 0.01	0.71 ± 0.04	0.66 ± 0.02	0.64 ± 0.05	0.68 ± 0.01	0.73 ± 0.01
TAFE4_mic	0.67 ± 0.01	0.76 ± 0.03	0.65 ± 0.02	0.58 ± 0.04	0.70 ± 0.01	0.71 ± 0.01
TAFE4_ast	0.62 ± 0.04	0.75 ± 0.15	0.65 ± 0.03	0.44 ± 0.14	0.69 ± 0.06	0.65 ± 0.04

As can be seen, the PathFinder can successfully classify the majority of cells in the test dataset into the correct condition. This means that, after training, the model learned the most important difference between the two conditions from a huge gene expression profile. Such differences can be reflected in the important score of each path, as the final prediction is made based on the different predefined paths. Among all results, the standard deviation of the metrics for TAFE4_ast is much larger than the other two cell types. We speculate that this discrepancy is caused by the limited number of cell samples in the TAFE4_ast group, which makes the model easily overfit to the training data.

Then, we evaluate the learned path score from each group. For each cell group, we first average the learned path score from five repeated runs to get the final path score. We average the absolute fold-change level of all genes within each path to get an average differential expression level for each path. Then, we compare the top 200 selected paths from the results of the PathFinder model to the remaining paths. The results are shown in Figure 2B. We can see that, for all three different cell types, the selected top 200 paths from PathFinder have a much higher average differential expression level compared to the remaining paths. The results indicate that PathFinder is effective in ranking differential expressed paths through the training. This can be attributed to two objective functions used in PathFinder. First, by minimizing the classification loss, the model is forced to increase the score for paths that are useful for separating two different conditions. It is intuitive that paths with higher average differential expression levels are more helpful for the prediction. Second, by minimizing the regularization loss, the model tends to give a high score for paths with high prior weight, and the prior weight is positively related to the average differential expression level.

Then, we evaluate the robustness and stability of the PathFinder. Concretely, we want the final path score distribution (ranking) learned from PathFinder to be stable and robust even if we slightly alter the training data. Since we randomly split the whole dataset for each repeated run, we can directly compare the learned score for each run to achieve our goal. Therefore, we plot the learned score for all paths, and all runs with paths are sorted by the average score. The results are shown in Figure 2C. For all three cell types, the learned scores are very stable across different runs, as paths with higher ranks always have higher scores. This means that, even if we slightly alter the training dataset, the PathFinder model can still output almost the same top k paths. The results successfully demonstrate the robustness of the PathFinder model for extracting important paths and constructing intra-communication networks.

Finally, we further evaluate the effectiveness of PathFinder on intra-cell signaling networks using the human cirrhosis cohort. Specifically, we run PathFinder on endothelial, macrophages, and T cells. The procedure is the same as the AD cohort. The average evaluation metric on the test set can be found in Supplementary Table S2 and the comparison of the average differential expression level of paths can be found in Supplementary Figures S1A,B.

### Core intra-cell signaling networks associated with the APOE4 genotype

**Figure 3:**
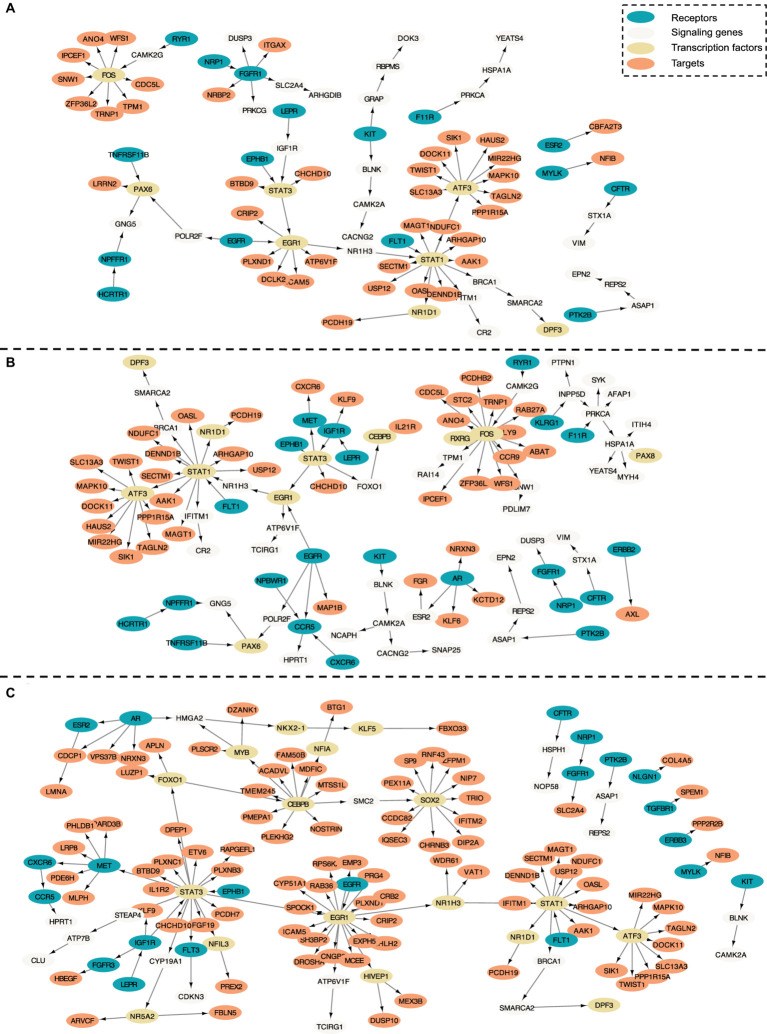
Intra-cell communication networks discovered by the PathFinder model for the AD cohort. **(A)** Excitatory neurons; **(B)** Microglia; **(C)** Astrocyte.

**Figure 4:**
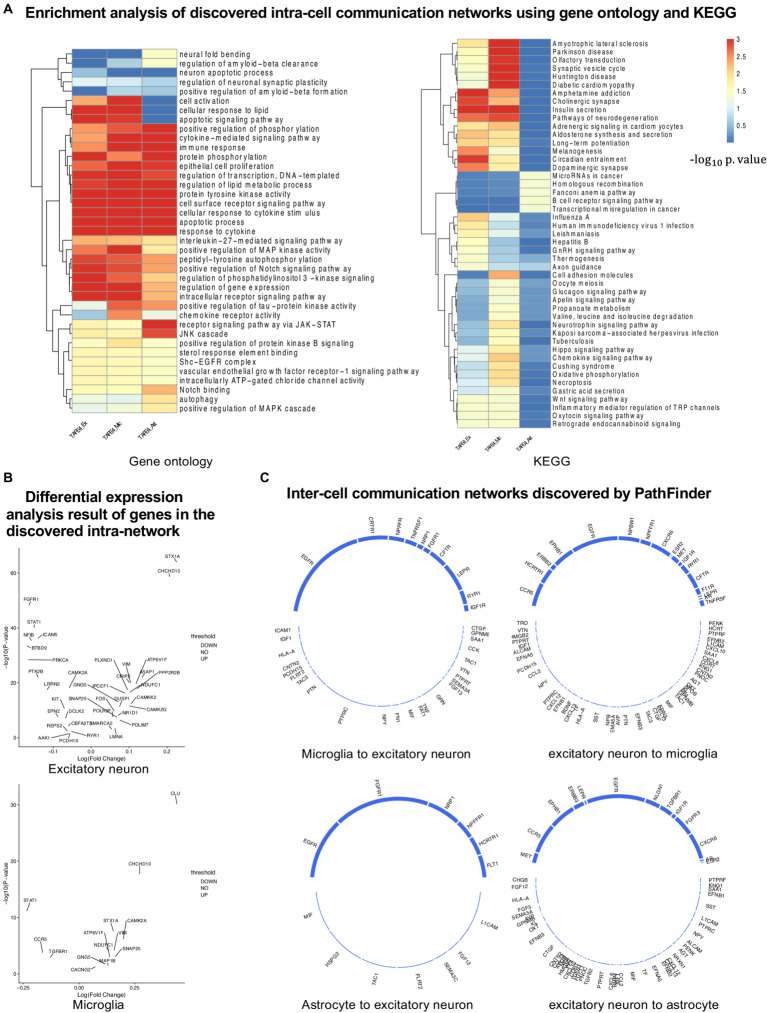
Analyses of the results. **(A)** KEGG and GO enrichment analyses on all discovered intra-networks. **(B)** Differential expression analysis. **(C)** Inter-cell communication networks. All ligands are from DEGs of the ligand cells. Receptors are marked as blue.

In this section, we evaluate the intra-cell communication networks discovered by the PathFinder model. Particularly, we want to know whether the discovered networks can reveal the recent discovery of APOE4-driven AD or even indicate new findings. First, for all three cell types, the final networks are generated by first averaging the path score learned from five repeated runs and then ranking and selecting the top 300 paths from all paths to form the final networks. The generated networks for all three cell types are shown in Figure 3. Then, we perform the enrichment analysis on all generated networks using KEGG signaling pathways and gene ontology (GO) terms. The enrichment results are shown in Figure 4A. Based on the results, we find several key factors that are important to the development of APOE4-driven AD.

#### Neuron inflammation

Numerous studies have shown that inflammation is highly activated and plays a key role in the progress of AD (Rogers et al., 1996; Akiyama et al., 2000; Halliday et al., 2000; Mathys et al., 2019). From the enrichment results, we can see that many inflammation-related pathways/GO terms are enriched across multiple cell types. For example, *cytokine-mediated signaling pathway, cellular response to cytokine stimulus,* and *inflammatory mediator regulation of TRP channels*. This result aligns with the findings of previous studies and further confirms that the existence of APOE4 in the astrocyte stimulates the inflammatory response. More specifically, several genes related to neuron inflammation are identified by PathFinder across multiple cell types. STAT1 and STAT3 are identified as hub genes connected to multiple targets in both the network of neurons and microglia. It has been shown that STAT1 plays a key role in regulating inflammatory responses and cellular death (Hu et al., 2002; Butturini et al., 2018). Moreover, the differential expression analysis (Figure 4B) reveals that STAT1 is highly differentially expressed in the TAFE4 group, which further confirms the important role of STAT1.

#### Autophagy

In addition to inflammation, the *Apoptotic* and *Apoptotic signaling pathways* are enriched in the neuron and the microglia. Autophagy is a lysosome-dependent, homeostatic process, in which organelles and proteins are degraded and recycled into energy. Autophagy has been linked to Alzheimer’s disease pathogenesis through its merger with the endosomal-lysosomal system, which has been shown to play a role in the formation of the latter amyloid-β plaques (Funderburk et al., 2010). One hypothesis states that irregular autophagy stimulation results in increased amyloid-β production (Yu et al., 2005). The existence of APOE4 may also affect the process of autophagy, leading to the accumulation of amyloid-β in the brain affected by AD. Particularly, CLU and FOXO1 genes are identified in the intra-network of microglia and astrocytes. CLU is one of the top AD candidate genes. Some study shows that it is a causal gene of AD-affected hippocampal connectivity (Zhang et al., 2015). Moreover, it is shown that CLU protein interacts with Aβ, reduces its aggregation, and protects against its toxic effects (Beeg et al., 2016). Many studies have shown that FOXO1 induces autophagy in cardiomyocytes and cancer cells. FOXO1 has been identified as a gene that encodes for a transcription factor involved in modulating autophagy in neurons (Xu et al., 2011).

#### Lipid transportation

*The regulation of lipid metabolic process and cellular response to lipids* are enriched in the intra-communication network of all three cell types. The enriched genes included NR1D1, EGR1, and BRCA1. It has been proved that APOE4 is involved in the lipid transportation and metabolism (Tindale et al., 2017). The existence of APOE4 in the astrocyte may disturb the brain lipid composition and thus affect the blood–brain barrier (BBB) function (Chew et al., 2020). All these results confirm the influence of APOE4 in the progress of AD and the dysfunction and death of the neuron.

#### JAK-STAT signaling pathway

In the intra-communication network of the astrocyte, the receptor signaling pathway via JAK–STAT is enriched with the corresponding gene: STAT3, SOCS3, HMGA2, and STAT1. The JAK–STAT signaling pathway has been reported to be the inducer of astrocyte reactivity (Ben Haim et al., 2015). The enrichment of the pathway indicates that the existence of APOE4 in astrocytes can influence the function of the JAK–STAT signaling pathway, and the pathway reversely affects the activity of the astrocyte.

### Evaluation of the intra-cell signaling networks on human cirrhosis

**Figure 5:**
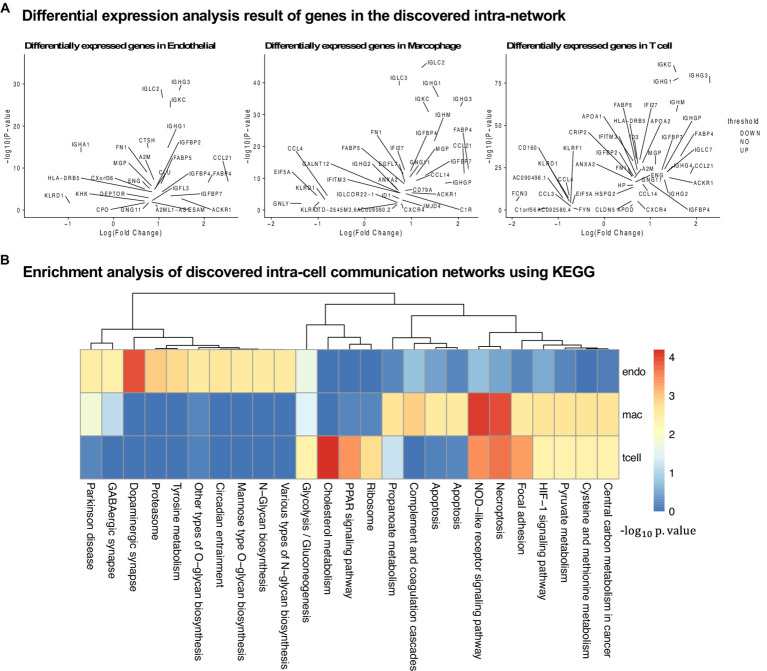
Analysis of the results for the cirrhosis cohort. **(A)** Differential expression analysis for all three cell types. **(B)** KEGG pathway enrichment analysis using the intra-cell networks discovered by PathFinder.

**Figure 6:**
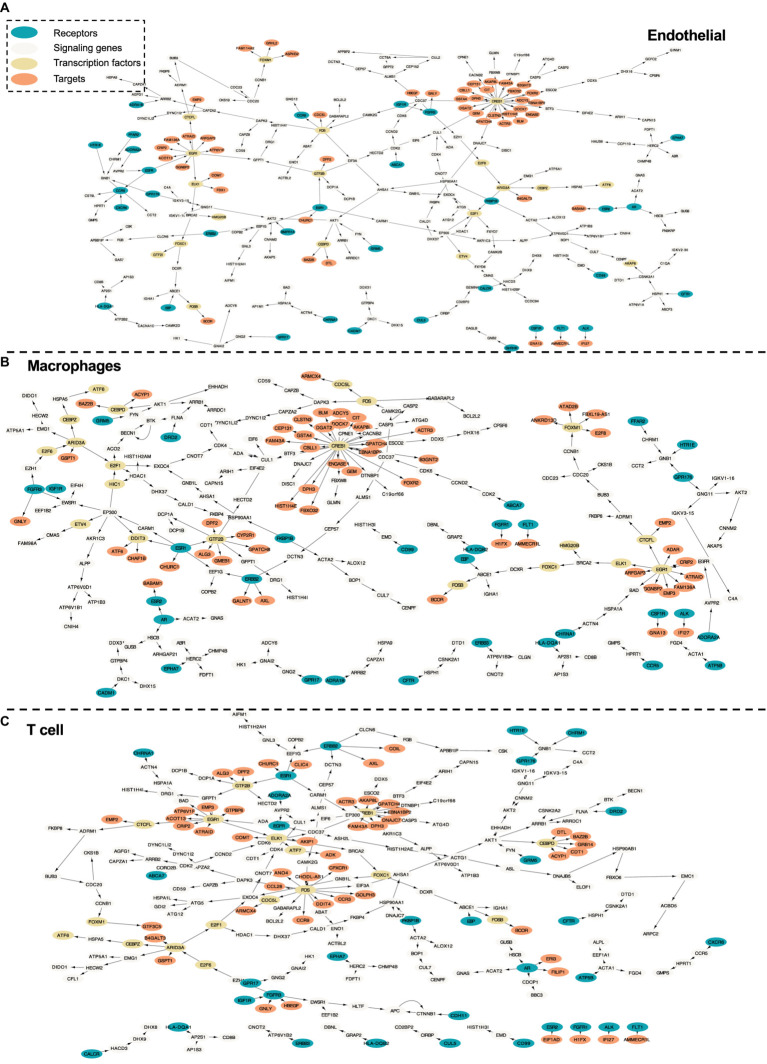
Intra-cell communication networks discovered by the PathFinder model for the human cirrhosis cohort. **(A)** Endothelial; **(B)** Macrophages; **(C)** T cell.

In this section, we further evaluate the intra-cell signaling networks on human cirrhosis on endotheilal, marcrophages, and T cells. The network extraction procedure is the same as the AD cohort. The gene expression and the pathway enrichment analysis result are shown in Figure 5. The final intra-networks for each cell type are shown in Figure 6. Before the analysis, we compare the extracted intra-cell network of cirrhosis with that obtained from the AD cohort. We merge the genes from all three cell types together for AD and cirrhosis separately and then compare the common genes from both cohorts. There are 269 genes from cirrhosis and 110 genes from AD. However, there are only 14 common genes, which demonstrate that PathFinder is disease- and expression-specific. We further explore the networks identified by the PathFinder model and their relationship with cirrhosis.

#### The role of immune cells in liver diseases

Immune cells and various signaling pathways play an important role in the pathogenesis of liver diseases. Gene CCR9 is activated in the intra-cell signaling network of both endothelial and T cells. Studies have found that, in a mouse model of NASH, the CCR9/CCL25 axis promotes the recruitment of macrophages and the formation of fibrosis, providing a new potential therapeutic target for NASH (Morikawa et al., 2021). On the other hand, liver NKT cells accumulate in a CXCR6-dependent manner early after injury, exacerbating the inflammatory response and promoting the progression of liver fibrosis, suggesting that the CXCR6/CXCL16 pathway may be an effective target for the treatment of liver fibrosis (Wehr et al., 2013). CXCR6 is discovered by PathFinder for the intra-cell signaling network of both endothelial and macrophages, which further confirms it. Additionally, β-arrestin1 (ARRB1) activated at the signaling network of all three cell types was reported to interact with pro-GDF15, promoting its cleavage and maturation in the Golgi apparatus, and the absence of ARRB1 significantly exacerbates hepatic steatosis, fibrosis, and inflammation (Zhang et al., 2020).

#### Liver fibrosis and its reversibility

The development of liver fibrosis is a complex and potentially reversible process. In its early stages, liver fibrosis may not immediately present severe symptoms but can eventually progress to cirrhosis and affect multiple organs. CREB is a highly activated gene discovered by PathFinder. Research has found that CREB, a molecule downstream of the cAMP signaling pathway, can serve as a therapeutic target for fibrosis (Li et al., 2019). Furthermore, insulin-like growth factor 1 (IGF1) and its receptor IGF1R play a crucial role in liver health and function, primarily expressed in the liver tissue. Studies on liver fibrosis have revealed the core role of the IGF1/IGF1R signaling system in controlling the liver fibrosis process (Gui et al., 2023). In the intra-cell signaling network of all three cell types identified by PathFinder, IGF1R is activated and further triggers target GNLY and HBEGF through FGFR3. Although there is not enough literature discussing their relationship with cirrhosis, exploiting the molecular mechanisms and functionality may provide new insights into studying cirrhosis and be helpful in developing more effective treatments to solve liver disease problems.

#### Liver disease transition process

In the intra-cell signaling networks identified by PathFinder, genes EGR1 and ERBB3 are highly activated. In the liver disease transition process, chronic hepatitis and cirrhosis are major factors leading to the majority of hepatocellular carcinomas (HCC). Concurrently, non-alcoholic fatty liver disease (NAFLD) has become a global epidemic, not only associated with the development of metabolic syndrome but also regarded as a pathway leading to severe liver diseases such as cirrhosis and hepatocellular carcinoma. In this transition process, EGR1 has been discovered as a key regulator of NAFLD, presenting potential as a potent target for intervening in NAFLD (Guo et al., 2023). Additionally, research has identified ERBB3 as a potential serum marker for early HCC in patients with chronic hepatitis and cirrhosis (Nasiri et al., 2020). A deeper understanding of the mechanisms underlying liver disease transition will provide insights into therapeutic strategies for related diseases.

### Core multi-cell inter-cell communication networks associated with the APOE4 genotype

To further understand the complex signaling flow and mechanism behind the APOE4 and AD pathology, we further generate inter-cell communication networks between three different cell types using PathFinder, as shown in Figure 4C. First, we can see that, compared to astrocytes, microglia have much more interactions with neurons. This may indicate that the existence of APOE4 in the astrocyte may activate the functionality of microglia and then cause abnormal activities in the neurons. Among all interactions, several interesting interactions appealed to the result. First, the MIF secreted by the astrocyte interacts with the EGFR in the neuron and follows downstream signaling. The MIF is a well-known proinflammatory cytokine that promotes the production of other immune mediators. Increased expression of MIF can contribute to chronic neuroinflammation and neurodegeneration (Tavassoly et al., 2020). EGFR is a potential target for treating AD-induced memory loss (Zhu et al., 2011; Wang et al., 2012). The increased expression level of MIF could be the signature of activated astrocytes, and the MIF further triggers the expression of EGFR and the subsequent downstream network in the neuron, which contributes to neuron inflammation and degeneration.

In addition to MIF in astrocytes, many ligands for receptor EGFR are also identified in microglia, including ICAM1, IGF1, HLA-A, CNTN2, PCDH15, FLRT2, TAC3, PTN, and PTPRC. The downregulation of PTPRC is reported to contribute to the overproduction of Aβ and neuron loss (Brito-Moreira et al., 2017). Another interaction is the *NLGN1* gene which is expressed in neurons that interact with the *NRXN1* gene in the astrocyte. The amyloid-β oligomers are synaptotoxins that build up in the brains of patients and are thought to contribute to the memory impairment in AD. It has been shown that the interaction of neurexins (Nrxs) and neuroligins (NLs) is critical for synapse structure, stability, and function (Tyzack et al., 2017). The dysregulation of the interaction between Nrxs and NLs may contribute to the formation of amyloid-β oligomer. The *EFNA5* in the neuron is upregulated in the neuron and interacts with *EPHB1* and downstream *STAT3* signaling in the astrocyte. This interaction is closely related to the ephrin-B1-mediated stimulation. The analysis has shown that the ephrin-B1-mediated stimulation induces a protective and anti-inflammatory signature in astrocytes and can be regarded as “help-me” signal of neurons that failed in early amyotrophic lateral sclerosis (ALS) (Lambert et al., 2018). Such signals could also play an important role in triggering inflammation and neuron degeneration in the CNS system.

## Conclusion and discussion

In this study, we propose PathFinder, which is the first deep-learning model with a graph transformer that can be used to extract both intra- and inter-cell communication networks using scRNA-seq data. Through a case study using an AD scRNA-seq dataset from mice, we evaluate the effectiveness of PathFinder from multiple perspectives. First, the quantitative analysis confirms that PathFinder performs well in separating cells from different conditions by leveraging the difference of expression patterns in the signaling paths. Furthermore, the learned path score is robust and consistent in repeat runs. We further evaluate the correctness of extracted networks through extensive literature searches. The resulting network aligned well with many recent discoveries on the AD pathology, which further proved the effectiveness of the proposed PathFinder. Additionally, the current version of PathFinder has a few potential limitations to be improved in the future studies. First, it requires many samples in training to produce reasonable results. Second, it relies on the pre-defined paths from the database to learn and extract meaningful patterns and is unable to discover new signaling flows. Third, currently, it is hard to validate the discovered signaling pathway quantitatively as there is no existing benchmark for conducting this process. All these limitations warrant further investigation. For example, we can construct a common benchmark to evaluate the performance of all signaling network inference methods quantitatively. We will also improve the model in our future work.

## Methodology

### Gene-gene interaction database collection and processing

To construct the gene–gene interaction database, the raw interaction data were collected from NicheNet software (Browaeys et al., 2020). The raw interaction data were divided into three types: ligand-receptor network, signaling network, and gene-regulation network. The original network contained 12,019 interactions/1,430 genes, 12,780 interactions/8,278 genes, and 11,231 interactions/8,450 genes, respectively. To construct the intra- and inter-network database, the data were further processed by the following steps.

First, ligands and receptors were collected by gathering the source and target of the ligand-receptor network. There were a total of 688 ligands and 857 receptors. Then, interactions in the ligand-receptor network were divided into two types. If one interaction exists in both directions in the database, we labeled it as bidirectional. Otherwise, we labeled it as directional. After processing, there were 11,880 directional interactions and 139 bidirectional interactions.

The gene-regulation network was processed as follows. First, 1,639 transcriptional factors (TFs) were collected (Fan et al., 2021). For convenience, TFs that exist in either the ligand or receptor list were removed. Finally, 1,632 TFs were collected. Then, three different types of regulation were collected in the gene-regulation interaction network, which are ligand regulation, receptor regulation, and TF regulation. To label each interaction into one of three types, all the interactions in the network were removed if the source gene was not in the ligand, receptor, or TF list. Then, the interactions were labeled based on the type of source (e.g., if the source of interaction is a receptor, we label it as receptor regulation). After processing, there were 1,329 ligand-regulation interactions, 272 receptor-regulation interactions, and 6,706 TF-regulation interactions.

Finally, the signaling network was processed as follows. First, all interactions were removed if they existed in either the ligand-receptor or the gene-regulation network. Then, the interactions were further divided into receptor-TF, receptor-signaling, signaling-TF, and signaling-signaling. To be more specific, if the source of interaction is in the receptor list and the target of interaction is in the TF list, the interaction was labeled as receptor-TF. If the source of interaction is in the receptor list and the target is not in the tTF list, the interaction was labeled as receptor-signaling. If the source of interaction is not in the receptor list and the target of interaction is in the TF list, the interaction was labeled as signaling-TF. If neither the source nor target of interaction is in the TF and receptor lists, the interaction was labeled as signaling-signaling. The interactions that cannot be classified into one of the specified groups were removed for convenience. Finally, there are 31 receptor-TF interactions, 524 receptor-signaling interactions, 975 signaling-TF interactions, and 9,745 signaling-signaling interactions.

### Notations and terminologies

#### Terminologies

An embedding or a representation is a vector of size $R^{d}$ that represents an entity, such as a gene or a path. The input embedding is the embedding input to the model, the hidden embedding is the embedding output by the middle layers of the model, and the output embedding is the embedding output by the model. With the final output embedding for an entity, we can do the classification or regression by passing it to a logistic regression or linear regression layer. An encoding is a function that transforms an entity to the embedding. Typically, the goal of a deep learning or machine learning model is to learn a model that can take the input embedding of the entity we want to predict and output the output embedding which is more reliable and powerful for the prediction. A single neural network layer will contain one or multiple trainable weight matrices. These matrices is responsible for transforming the input embedding into the output embedding. They will be updated and refined by the backward propagation and gradient descent used in the neural network.

#### Notations

A gene graph is denoted as G=(V,E) , where V is the set of gene nodes with |V|=n , E is the set of edges and E⊆V×V . The node embedding set is denoted by $X={\left[x_{1},x_{2},\ldots ,x_{n}\right]}^{T}\in R^{n\times d}$ , where $x_{u}\in R^{d}$ is the embedding vector of the node u . The graph structure is defined by an adjacency matrix $A\in {\left[0,1\right]}^{n\times n}$ , where $A_{uv}=1$ indicate there is an edge from the node u to node v and $A_{uv}=0$ otherwise. Furthermore, a set of paths sampled from a graph is denoted as $P=\left\{p_{1},\;p_{2},\ldots ,\;p_{p}\right\}$ , where $p_{m}$ is the m -th path, which is a list to store the nodes of the path in order. Paths can have different lengths, and we denote the length of path m be $l_{m}$ .

### Preliminary of transformer and Graphormer

The transformer is a powerful architecture in the deep learning field. It consists of multiple transformer layers. Each transformer layer has two parts: a multi-head self-attention and a point-wise feed-forward network (FFN) with residual connection applied between each part. Let $H^{l-1}\in R^{n\times h_{emb}}$ be the embedding of nodes in layer l−1 , and $H_{u}^{l-1}$ is the embedding of the node u in layer l−1 , the computation of multi-head self-attention is:


$$Q^{l,i}=H^{l-1}W_{Q}^{l,i},K^{l}=H^{l-1}W_{K}^{l,i},V^{l}=H^{l-1}W_{V}^{l,i},$$

$$head_{i}=Attention\left(Q^{l,i},K^{l,i},V^{l,i}\right)=SoftMax\left(\frac{Q^{l,i}K^{l,iT}}{\sqrt{d_{k}}}\right)V^{l,i},$$

$$O^{l}=Concat\left(head_{1},\ldots ,head_{h}\right)W_{O}^{l},$$

where $W_{Q}^{l,i},W_{K}^{l,i},W_{V}^{l,i}\in R^{h_{emb}\times d_{k}}$ , and $W_{O}^{l}\in R^{hd_{k}\times h_{emb}}$ are all trainable weight matrix, h is the number of heads, $O^{l}\in R^{n\times h_{emb}}$ is the output from the multi-head self-attention in layer l , Concat is the concatenation function to combine multiple vectors into one single large vector. For simplicity, we let $h\times d_{k}=h_{emb}$ . The output $O^{l}$ will then be fed into a point-wise feed-forward network. The computation of the point-wise feed-forward network is:


$$FFN\left(x\right)=ReLu\left(xW_{1}^{l}+b_{1}^{l}\right)W_{2}^{l}+b_{2}^{l},$$

where $W_{1}^{l}\in R^{h_{emb}\times h_{emb}},W_{2}^{l}\in R^{h_{emb}\times h_{emb}}$ , $b_{1}^{l}\in R^{2h_{emb}}$ , and $b_{2}^{l}\in R^{h_{emb}}$ are all trainable weight matrix and bias. Notice that here we slightly modify the hidden size of the feed-forward network of the original model. The embedding of each node $O_{i}^{l}\in R^{h_{emb}}$ will be input into this FFN for further processing.

However, the vanilla transformer cannot be used directly on the graph structure data as it lacks a critical part for encoding the topological information into the model. To deal with this issue, Graphormer proposed several novel encodings into the model. Specifically, they introduced centrality encoding, spatial encoding, and edge encoding. The centrality encoding is used to embed the graph centrality information into the model. Given the input data X , the computation of centrality encoding is:


$$H^{0}=X+Z^{-}\left\{deg^{-}\left(G\right)\right\}+Z^{+}\left\{deg^{+}\left(G\right)\right\},$$

where the $Z^{-},Z^{+}$ are all trainable embedding vectors and $deg^{-}\left(G\right)$ , $deg^{+}\left(G\right):G\to R^{n}$ are the function to compute the in-degree and out-degree of each node in the graph G . The spatial and edge encoding is used to encode the graph structure into the model. With the spatial and edge encoding, the self-attention is revised as:


$$head_{i}=SoftMax\left(\frac{Q^{l,i}K^{l,iT}}{\sqrt{d_{k}}}+b_{i}\left\{\phi \left(G\right)\right\}+c_{i}\right)V^{l,i},$$

where $b^{i}$ is trainable embedding vectors to encode the spatial information at head i and $\phi \left(G\right):G\to R^{n\times n}\;$ is the function to compute the shortest path length between each two nodes. If two nodes are not connected, a special value will be used. $c^{i}\in R^{n\times n}$ is the edge embedding and $c_{uv}^{i}=\frac{1}{N}\overset{N}{\underset{n=1}{\sum }}x_{en}w_{n}^{iT}$ , where $x_{en}$ is the edge feature of the n -th edge in the shortest path between node u and node v and the $w_{n}^{i}$ is trainable weight vector of n -th edge of head i . Note that both the spatial and edge encodings are unique across different layers.

### Architecture of PathFinder

The PathFinder model consists of three components, namely, the node encoder, path encoder, and graph encoder. The overall architecture of the PathFinder model is shown in Figure 1, lower. The rationale behind PathFinder is that, if a model can identify disease cells from normal cells, it must learn useful knowledge from the gene expression profile to help it make that prediction. In PathFinder, we introduce the path encoder to let the model make the prediction based on the importance of the signaling paths with their corresponding expression. In this way, if the model can make a reasonable prediction, it must have the ability to distinguish differential expressed signaling paths from the other paths, and that is exactly what we are looking for. Furthermore, since the paths are pre-defined from the physical interaction database in a biologically meaningful way, the extracted signaling paths are inherently biologically meaningful. PathFinder can be seen as a simulator to simulate the signaling path in the cell and use it to make the prediction. Below, we discuss each component in detail.

#### Node encoder

The architecture of the node encoder is similar to the Graphormer, which stacks L transformer layer with centrality encoding, spatial encoding, and edge encoding. The input to PathFinder is the expression value of each gene in a cell sample. However, we made several modifications to the original architecture. First, the hidden size in the point-wise feed-forward network is all $h_{emb}$ in both two layers for simplicity. Second, the edge encoding in PathFinder is modified. In the original Graphormer, the edge encoding is computed by all the edges in the shortest path between two nodes, which can capture long-range information in the graph. However, the localized feature in the graph will be smoothed in such a manner. Instead, PathFinder aims for the node embedding learned from the node encoder to focus on the localized information in the graph. Therefore, direct edge encoding is proposed. The direct edge encoding is computed by:


$$c_{uv}^{i}=x_{uv}w^{iT}$$

Where $x_{uv}$ is the edge feature of the edge between node u and node v . If there is not an edge between two nodes, the direct edge encoding is set to a special vector for simplicity. By doing this, the node encoder becomes adept at learning node embedding that capture localized information. Finally, the spatial encoding is also revised in PathFinder. Since here the graph structure is identical for all samples and the node order invariant is automatically held, we can learn a specific spatial encoding for each pair of two nodes. Therefore, we design the node index encoding in the PathFinder model. The node index encoding is not computed from the length of the shortest path between each pair of nodes but is directly learned for each pair of two genes, namely, for each pair of two genes, a unique encoding is learned for each head in each layer of the node encoder.

#### Path encoder

Furthermore, the path encoder is responsible for learning gene signaling path embedding, utilizing the node embedding in the graph and the pre-defined path list of the graph. The details of the pre-defined path list are illustrated below. Suppose there are p unique paths in the path list P , where the length of the m -th path is $l_{m}$ and the total number of nodes in the path list is k (count repeated nodes in different paths). Denote the node embedding output from the layer l as $H^{l}$ , we first learn a path-specific embedding through:


$$U_{i}^{l}=scatter{\left(H^{l}\right)}_{i}W_{u}^{l}+b_{u}^{l},$$

where $W_{u}^{l}\in R^{h_{emb}\times u}$ and $b_{u}^{l}\in R^{u}$ are all trainable weight matrix, scatter is a function to reorder and scatter the node in the graph into the order of the pre-defined path list. For example, suppose there are five embedding genes output from the node encoder. That is $H^{l}\in R^{5\times h_{emb}}$ . We label each gene from 1 to 5. Suppose there are two paths. The first path is 1- > 3- > 4. The second path is 2- > 3- > 4- > 5. Then, the $scatter\left(H^{l}\right)$ will output a new matrix with the size of 7 and each row represents a gene in a path. For instance, the third row is $H_{4}^{l}$ since it is the third gene in the first path. $U^{l}\in R^{k\times u}$ is the learned path-specific embedding. For convenience, we denote $U_{m,i}^{l}$ as the embedding of i -th node in the m -th path. Then, path positional and path edge encodings are introduced to encode additional information for all paths. Let ${\bar{U}}^{l}$ be the result embedding after the special encodings. We have:


$${\bar{U}}_{m,i}^{l}=U_{m,i}^{l}+p_{i}^{l}+e_{i,i+1}^{l},$$

Where $p_{i}^{l}$ is the learnable positional encoding vector and its value only depends on the position i , $e_{i,i+1}^{l}$ is the learnable edge encoding to encode the edge type between i -th node and i+1 -th node. Then, the score of each node within the path is computed by:


$$S_{m,i}^{l}=\tanh\left({\bar{U}}_{m,i}^{l}W_{s1}^{l}+b_{s1}^{l}\right)W_{s2}^{l}+b_{s2}^{l}$$

$${\bar{S}}^{l}=ScatterSoftMax\left(S^{l}\right),$$

where $W_{s1}^{l}\in R^{u\times r}$ , $b_{s1}^{l}\in R^{r}$ , $W_{s2}^{l}\in R^{r\times r}$ , and $b_{s2}^{l}\in R^{r}$ are all trainable parameters. ScatterSoftmax is the softmax function working within each path. The $S^{l}\in R^{k\times r}$ is the final r set important score for each node in each path. We let $r\times u=h_{emb}$ for simplicity. After we obtain $S^{l}$ , the path embedding is computed by:


$$P^{l}=Flatten\left(ScatterSum\left(S^{l}*{\bar{U}}^{l}\right)\right)$$

∗ is the point-wise product working on each set of important scores. That is, for each set of important scores, we do a point-wise product of that set of scores and $U^{l}$ , which results in total r sets. The ScatterSum function is the summation on each path. Flatten is the function to flatten the embedding of all sets. $P^{l}\in R^{p\times h_{emb}}$ is the final path embedding in the layer l .

#### Graph encoder

In the original Graphormer, the graph embedding is learned by introducing a special node and letting it connect to all the nodes in the graph. After forwarding, the embedding of that special node is regarded as the graph embedding for the graph-level task. In PathFinder, our goal is to learn the graph embedding from the path embedding. Meanwhile, we aim to extract the important paths from the model after training it for the graph-level task. To simultaneously achieve both goals, the graph encoder is proposed. The graph encoder consists of two parts. The first part is a trainable path weight and the sigmoid function to assign each path with different scores. The second part is the jumping knowledge network to combine the graph embedding in each layer and compute the final embedding.

In PathFinder, the graph embedding is learned by integrating all the path embeddings from each layer, which requires an important score for each path. Normally, the score is computed based on one sample. However, such a score is not robust and may vary a lot even with a minor variation of the path embedding (Xu et al., 2018; Chen et al., 2019; Fan et al., 2021). To avoid the issue and learn a robust important score across the whole dataset, the trainable path score $M\in R^{p}$ is introduced. M is identical to all samples and layers and learned through backpropagation. The path important score is computed by:


I=Sigmoid(M),

where $I\in R^{p}$ is the important score for each path. Then, the graph embedding of layer l is computed by:


$$g^{l}=IP^{l},$$

where $g^{l}$ is the graph embedding of layer l . The final step of the graph encoder is to integrate the graph embedding of each layer and learn a final embedding. Here, we utilize the idea of JumpingKnowledge network (Xu et al., 2018) and compute the final graph embedding by:


$$G=MaxPooling\left(Concat\left(g^{1},g^{2},\ldots ,g^{L}\right)\right),$$

where MaxPooling is the max pooling function and $G\in R^{h_{emb}}$ is the final graph embedding learned by PathFinder. Finally, the graph embedding is used to classify the cell sample into the corresponding condition (control/test). The prediction is a typical binary prediction computed by:


$$p=SoftMax\left(GW_{p}\right),$$

Where $W_{p}\in R^{h_{emb}\times 2}$ is the trainable projection matrix and p is the predicted distribution.

### Training and regularization of PathFinder

To train the PathFinder model, the negative log-likelihood (NLL) loss is applied. Let the $p_{i}^{c}$ be the predicted probability of the true condition of cell i , then the NLL loss is computed by:


$$L_{class}=\overset{N}{\underset{i=1}{\sum }}-\log\left(p_{i}^{c}\right)$$

Where the N is the number of cells in the dataset. Meanwhile, to regularize the training of the model and learn biological meaningful paths from the model, the regularization term is introduced to the path score M . Intuitively, the path that has a higher total fold change should have a higher path score. Furthermore, we designed three different regularization terms to generate different important paths by introducing the prior path score. Specifically, these three regularizations are upregulated path, downregulated path, and differentially expressed path regularization. Let the $fc_{i}^{m}$ be the log fold change of gene j in path m , then the prior path score is computed by:


$$S_{up}^{m}=Normalization\left(mean\left(\underset{j}{\sum }fc_{j}^{m}\right)\right)$$

$$S_{down}^{m}=Normalization\left(mean\left(\underset{j}{\sum }-fc_{i}^{m}\right)\right)$$

$$S_{\deg}^{m}=Normalization\left(mean\left(\underset{j}{\sum }|fc_{i}^{m}|\right)\right)$$

Where the $S_{up}^{m}$ , $S_{down}^{m}$ , and $S_{\deg}^{m}$ are the prior path scores for upregulated, downregulated, and differential expressed regularization, respectively. Normalization is the min-max normalization across all paths. Suppose we use the upregulated prior score, the regularization loss is computed by:


$$L_{reg}=D_{KL}\left(I∥S_{up}^{m}\right)$$

The final loss is:


$$L=L_{class}+\beta L_{reg}$$

Where β is the weight of the regularization term.

### Predefined path list

To train the PathFinder model, the path list needs to be defined before the training. Given the collected gene–gene interaction database and the input variable gene list, we designed several choices to generate a predefined path list. The first choice is the shortest path. For this choice, the shortest path between each pair of genes in the dataset will be computed and collected given the gene–gene interaction network. The second choice is to generate all the possible paths that start from the receptor and end in the target, which can also be performed using the gene–gene interaction database. To constrain the path, the minimum length of the path is set to be 3 unless the path is a receptor regulation interaction. The maximum length of the path is set to be 10.

### Experimental details

We conduct experiments to validate the effectiveness of PathFinder on TAFE_ex, TAFE_mic, and TAFE_ast cell sample datasets. For each dataset, we randomly split datasets into train/validation/test sets with a ratio of 0.7/0.1/0.2. We train the model using the train set and validate the performance of the model using the validation set. Finally, we save the model that achieves the best performance on the validation set and report the performance of the saved model on the test set. We use the area under the curve (AUC) as the performance metric for selecting the best model. We repeat experiments on each dataset five times (with a different random split applied to the dataset each time) and report the mean results and the standard deviation. The model and training hyperparameters are described as follows: We set the number of layers as 6 and the hidden size $h_{emb}$ as 128. The number of heads and scores set r as 8. For each experiment, we set the number of training epochs as 30, the learning rate as 0.0005, the dropout rate as 0.1, the regularization weight β as 0.1 for TAFE_ex and TAFE_mic, and 1.0 for TAFE_ast.

### Generation of the intra- and inter-cell communication network

After the PathFinder model is trained, the generation of an intra-cell communication network is straightforward. Concretely, we first average the path weight learned from five repeated experiments to get the final path weights. Furthermore, the top K paths are extracted and combined to generate the intra-cell communication network. The generation of the inter-cell communication network is as follows. Let the cell that provides ligands be the ligand cell and the cell providing receptors be the receptor cell. The intra-cell communication network is first generated. Then, the ligands of the ligand cell and receptors of the receptor cell will be extracted from their respective intra-networks. Then, the ligand–receptor pairs are selected given the ligand–receptor database. Finally, the kept pairs will be linked and the inter-network is generated.

## Data availability statement

Publicly available datasets were analyzed in this study. This data can be found at: https://www.ncbi.nlm.nih.gov/geo/query/acc.cgi?acc=GSE164507. The source code of PathFinder is publicly accessible on github: https://github.com/fuhaililab/PathFinder.

## Author contributions

JF: Data curation, Formal analysis, Methodology, Software, Visualization, Writing – original draft, Writing – review & editing. MP: Writing – review & editing. GL: Writing – review & editing. PP: Writing – review & editing. YC: Methodology, Writing – review & editing. FL: Conceptualization, Funding acquisition, Methodology, Writing – original draft, Writing – review & editing. HS: Writing – original draft, Formal analysis, Validation.

## Supplementary material

SUPPLEMENTARY Figure S1 Additional evaluation results. **(A)** Comparison of the differential expression level between paths identified by PathFinder and the rest in cirrhosis cohort. **(B)** The learned path scores of PathFinder on different runs on cirrhosis cohort.
