# Supplementary material for: PathFinder: a novel graph transformer model to infer multi-cell intra- and inter-cellular signaling pathways and communications
Source: Front Cell Neurosci. 2024 May 23;18:1369242. doi: 10.3389/fncel.2024.1369242 (PMC11155453; doi:10.3389/fncel.2024.1369242)

a. The comparison of the average differential expression level of paths extracted by PathFinder.

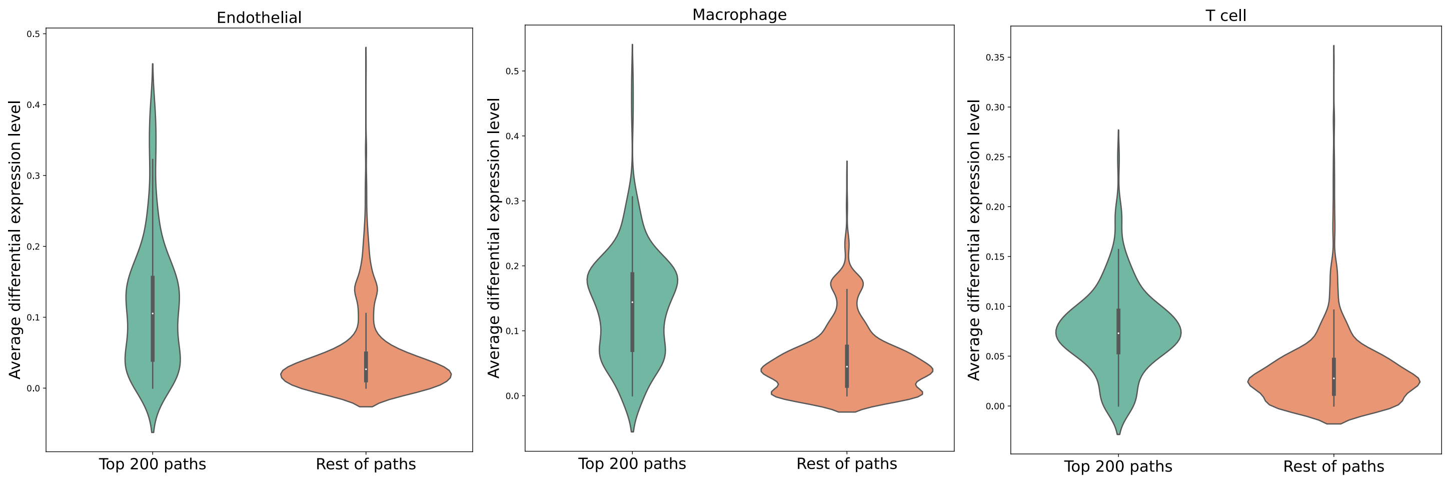

b. The learned path scores of PathFinder on different runs

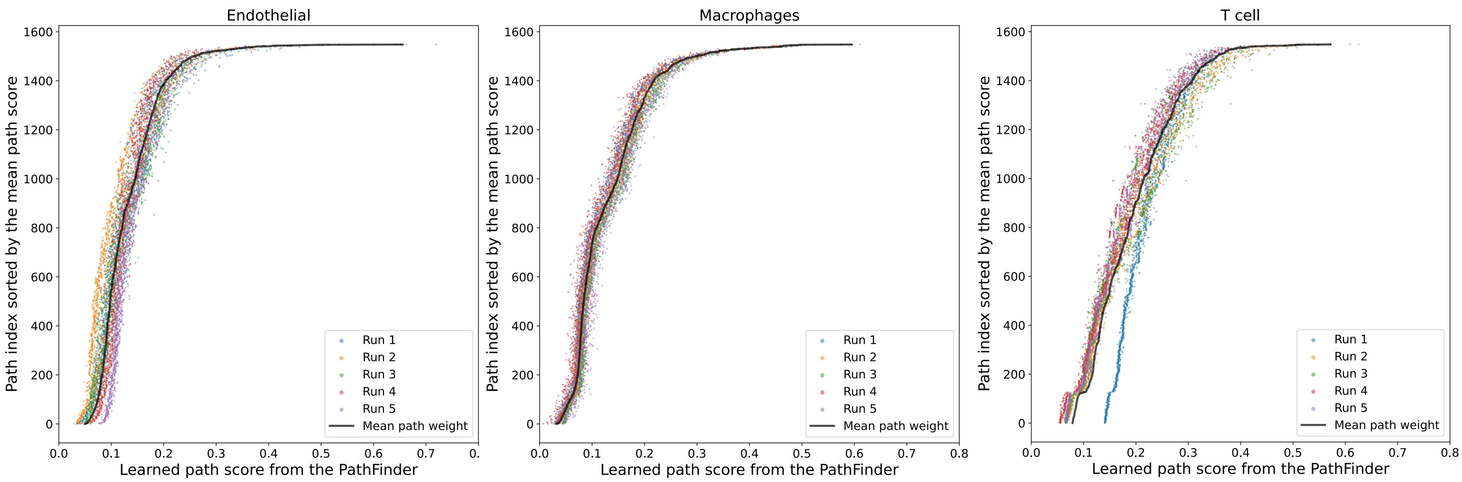

c. Comparison of the genes discovered by PathFinder for two cohorts.

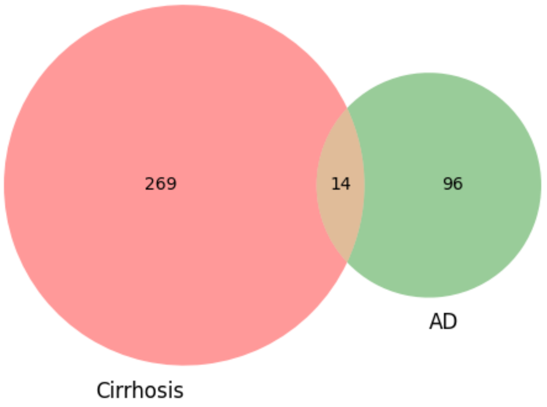

Supplement: SUPPLEMENTARY Figure S1 — Additional evaluation results. (A) Comparison of the differential expression level between paths identified by PathFinder and the rest in cirrhosis cohort. (B) The learned path scores of PathFinder on different runs on cirrhosis cohort. [file Figure_1.pdf]
