# Supplementary material for: PathFinder: a novel graph transformer model to infer multi-cell intra- and inter-cellular signaling pathways and communications
Source: Front Cell Neurosci. 2024 May 23;18:1369242. doi: 10.3389/fncel.2024.1369242 (PMC11155453; doi:10.3389/fncel.2024.1369242)
Supplement: Supplementary file 2 [file Data_Sheet_1.docx]

**Supplementary Table 1: The detailed cell distribution on mice AD and human cirrhosis cohorts.**

| **Cohort** | **Cell type** | **Control** | **Test** | **Total** |
| --- | --- | --- | --- | --- |
| **AD** | TAFE4_ex | 6857 | 6747 | 13604 |
|  | TAFE4_mic | 1930 | 1944 | 3874 |
|  | TAFE4_ast | 308 | 426 | 734 |
| **Cirrhosis** | Endothelial | 3924 | 2273 | 6197 |
|  | Macrophage | 5670 | 3503 | 9173 |
|  | T cell | 12834 | 8116 | 20950 |

**Supplementary Table 2: The detailed evaluation results on the test set for cirrhosis cohort.**

|  | Accuracy | Recall | Precision | Specificity | F1 | AUC |
| --- | --- | --- | --- | --- | --- | --- |
| Endo | 0.63±﻿0.07 | 0.84±﻿0.06 | 0.51±﻿0.06 | 0.52±﻿0.14 | 0.63±﻿0.03 | 0.75±﻿0.04 |
| Mac | 0.74±﻿0.06 | 0.83±﻿0.11 | 0.62±﻿0.06 | 0.68 ±﻿ 0.10 | 0.71±﻿0.06 | 0.85±﻿0.06 |
| Tcell | 0.63±﻿0.03 | 0.66±﻿0.05 | 0.48±﻿0.04 | 0.56 ±﻿ 0.18 | 0.56±﻿0.02 | 0.67±﻿0.03 |

**Supplementary Table 3: The top 100 paths discovered by the PathFinder on TAFE4_ex.**

| **Order** | **Path** |
| --- | --- |
| 0 | ['NRP1 -> FGFR1->PRKCG -'] |
| 1 | ['NRP1 -> FGFR1->DUSP3 -'] |
| 2 | ['NRP1 -> FGFR1->NRBP2 -'] |
| 3 | ['NRP1 -> FGFR1->NRBP2 -'] |
| 4 | ['NRP1 -> FGFR1->SLC2A4 -'] |
| 5 | ['NRP1 -> FGFR1->SLC2A4 -'] |
| 6 | ['NRP1 -> FGFR1->ITGAX -'] |
| 7 | ['NRP1 -> FGFR1->ITGAX -'] |
| 8 | ['FLT1 -> STAT1->NDUFC1 -'] |
| 9 | ['FLT1 -> STAT1->NDUFC1 -'] |
| 10 | ['FLT1 -> STAT1->NR1D1 -'] |
| 11 | ['FLT1 -> STAT1->NR1D1 -'] |
| 12 | ['FGFR1 -> NRBP2'] |
| 13 | ['FGFR1 -> ITGAX'] |
| 14 | ['FGFR1 -> SLC2A4'] |
| 15 | ['FLT1 -> STAT1->NR1D1 -> PCDH19 -'] |
| 16 | ['FLT1 -> STAT1->NR1D1 -> PCDH19 -'] |
| 17 | ['FLT1 -> STAT1->DENND1B -'] |
| 18 | ['FLT1 -> STAT1->DENND1B -'] |
| 19 | ['NRP1 -> FGFR1->SLC2A4 -> ARHGDIB -'] |
| 20 | ['FLT1 -> STAT1->USP12 -'] |
| 21 | ['FLT1 -> STAT1->USP12 -'] |
| 22 | ['FLT1 -> STAT1->AAK1 -'] |
| 23 | ['FLT1 -> STAT1->AAK1 -'] |
| 24 | ['FLT1 -> STAT1->ATF3 -'] |
| 25 | ['FLT1 -> STAT1->MAGT1 -'] |
| 26 | ['FLT1 -> STAT1->SECTM1 -'] |
| 27 | ['FLT1 -> STAT1->MAGT1 -'] |
| 28 | ['FLT1 -> STAT1->SECTM1 -'] |
| 29 | ['FLT1 -> STAT1->OASL -'] |
| 30 | ['FLT1 -> STAT1->OASL -'] |
| 31 | ['FLT1 -> STAT1->BRCA1 -'] |
| 32 | ['FLT1 -> STAT1->BRCA1 -'] |
| 33 | ['FLT1 -> STAT1->IFITM1 -'] |
| 34 | ['FLT1 -> STAT1->IFITM1 -'] |
| 35 | ['FLT1 -> STAT1->ARHGAP10 -'] |
| 36 | ['FLT1 -> STAT1->ARHGAP10 -'] |
| 37 | ['EGFR -> EGR1->NR1H3 -> STAT1 -> NDUFC1 -'] |
| 38 | ['EGFR -> EGR1->NR1H3 -> STAT1 -> NDUFC1 -'] |
| 39 | ['EGFR -> EGR1->NR1H3 -> STAT1 -> NR1D1 -> PCDH19 -'] |
| 40 | ['EGFR -> EGR1->NR1H3 -> STAT1 -> NR1D1 -'] |
| 41 | ['EGFR -> EGR1->NR1H3 -> STAT1 -> NR1D1 -'] |
| 42 | ['EGFR -> EGR1->NR1H3 -> STAT1 -> NR1D1 -> PCDH19 -'] |
| 43 | ['FLT1 -> STAT1->BRCA1 -> SMARCA2 -'] |
| 44 | ['EGFR -> EGR1->NR1H3 -> STAT1 -'] |
| 45 | ['FGFR1 -> SLC2A4->ARHGDIB -'] |
| 46 | ['EGFR -> EGR1->NR1H3 -> STAT1 -> DENND1B -'] |
| 47 | ['EGFR -> EGR1->NR1H3 -> STAT1 -> DENND1B -'] |
| 48 | ['IGF1R -> STAT3->EGR1 -> NR1H3 -> STAT1 -> NDUFC1 -'] |
| 49 | ['CFTR -> STX1A->VIM -'] |
| 50 | ['IGF1R -> STAT3->EGR1 -> NR1H3 -> STAT1 -> NDUFC1 -'] |
| 51 | ['IGF1R -> STAT3->EGR1 -> NR1H3 -> STAT1 -> NR1D1 -> PCDH19 -'] |
| 52 | ['IGF1R -> STAT3->EGR1 -> NR1H3 -> STAT1 -> NR1D1 -> PCDH19 -'] |
| 53 | ['EGFR -> EGR1->NR1H3 -> STAT1 -> USP12 -'] |
| 54 | ['IGF1R -> STAT3->EGR1 -> NR1H3 -> STAT1 -> NR1D1 -'] |
| 55 | ['EGFR -> EGR1->NR1H3 -> STAT1 -> USP12 -'] |
| 56 | ['IGF1R -> STAT3->EGR1 -> NR1H3 -> STAT1 -> NR1D1 -'] |
| 57 | ['EGFR -> EGR1->NR1H3 -> STAT1 -> AAK1 -'] |
| 58 | ['EGFR -> EGR1->NR1H3 -> STAT1 -> AAK1 -'] |
| 59 | ['EPHB1 -> STAT3->EGR1 -> NR1H3 -> STAT1 -> NR1D1 -> PCDH19 -'] |
| 60 | ['EPHB1 -> STAT3->EGR1 -> NR1H3 -> STAT1 -> NR1D1 -> PCDH19 -'] |
| 61 | ['EPHB1 -> STAT3->EGR1 -> NR1H3 -> STAT1 -> NDUFC1 -'] |
| 62 | ['EPHB1 -> STAT3->EGR1 -> NR1H3 -> STAT1 -> NDUFC1 -'] |
| 63 | ['RYR1 -> CAMK2G->FOS -> IPCEF1 -'] |
| 64 | ['RYR1 -> CAMK2G->FOS -> IPCEF1 -'] |
| 65 | ['FLT1 -> STAT1->ATF3 -> MIR22HG -'] |
| 66 | ['FLT1 -> STAT1->ATF3 -> MIR22HG -'] |
| 67 | ['EPHB1 -> STAT3->EGR1 -> NR1H3 -> STAT1 -> NR1D1 -'] |
| 68 | ['EPHB1 -> STAT3->EGR1 -> NR1H3 -> STAT1 -> NR1D1 -'] |
| 69 | ['FLT1 -> STAT1->ATF3 -> MAPK10 -'] |
| 70 | ['FLT1 -> STAT1->ATF3 -> MAPK10 -'] |
| 71 | ['PTK2B -> ASAP1->REPS2 -> EPN2 -'] |
| 72 | ['FLT1 -> STAT1->ATF3 -> DOCK11 -'] |
| 73 | ['FLT1 -> STAT1->ATF3 -> SLC13A3 -'] |
| 74 | ['FLT1 -> STAT1->ATF3 -> DOCK11 -'] |
| 75 | ['FLT1 -> STAT1->ATF3 -> PPP1R15A -'] |
| 76 | ['FLT1 -> STAT1->ATF3 -> SLC13A3 -'] |
| 77 | ['FLT1 -> STAT1->ATF3 -> SIK1 -'] |
| 78 | ['EGFR -> POLR2F->PAX6 -> GNG5 -'] |
| 79 | ['FLT1 -> STAT1->ATF3 -> TWIST1 -'] |
| 80 | ['FLT1 -> STAT1->ATF3 -> SIK1 -'] |
| 81 | ['FLT1 -> STAT1->ATF3 -> PPP1R15A -'] |
| 82 | ['EGFR -> POLR2F->PAX6 -> GNG5 -'] |
| 83 | ['FLT1 -> STAT1->ATF3 -> TAGLN2 -'] |
| 84 | ['FLT1 -> STAT1->ATF3 -> TWIST1 -'] |
| 85 | ['FLT1 -> STAT1->ATF3 -> TAGLN2 -'] |
| 86 | ['FLT1 -> STAT1->ATF3 -> HAUS2 -'] |
| 87 | ['FLT1 -> STAT1->ATF3 -> HAUS2 -'] |
| 88 | ['FLT1 -> STAT1->IFITM1 -> CR2 -'] |
| 89 | ['IGF1R -> STAT3->EGR1 -> NR1H3 -> STAT1 -'] |
| 90 | ['FLT1 -> STAT1->BRCA1 -> SMARCA2 -> DPF3 -'] |
| 91 | ['PTK2B -> ASAP1->REPS2 -'] |
| 92 | ['IGF1R -> STAT3->EGR1 -> NR1H3 -> STAT1 -> DENND1B -'] |
| 93 | ['IGF1R -> STAT3->EGR1 -> NR1H3 -> STAT1 -> DENND1B -'] |
| 94 | ['LEPR -> IGF1R->STAT3 -> EGR1 -> NR1H3 -> STAT1 -> NR1D1 -> PCDH19 -'] |
| 95 | ['LEPR -> IGF1R->STAT3 -> EGR1 -> NR1H3 -> STAT1 -> NR1D1 -> PCDH19 -'] |
| 96 | ['EGFR -> EGR1->NR1H3 -> STAT1 -> MAGT1 -'] |
| 97 | ['EGFR -> EGR1->NR1H3 -> STAT1 -> ATF3 -'] |
| 98 | ['EGFR -> EGR1->NR1H3 -> STAT1 -> SECTM1 -'] |
| 99 | ['EGFR -> EGR1->NR1H3 -> STAT1 -> MAGT1 -'] |
| 100 | ['EGFR -> EGR1->NR1H3 -> STAT1 -> OASL -'] |

**Supplementary Table 4: The top 00 paths discovered by the PathFinder on TAFE4_mic.**

| **Order** | **Path** |
| --- | --- |
| 0 | ['FLT1 -> STAT1->NDUFC1 -'] |
| 1 | ['FLT1 -> STAT1->NDUFC1 -'] |
| 2 | ['FLT1 -> STAT1->USP12 -'] |
| 3 | ['FLT1 -> STAT1->USP12 -'] |
| 4 | ['FLT1 -> STAT1->ATF3 -> MAPK10 -'] |
| 5 | ['FLT1 -> STAT1->ATF3 -> MAPK10 -'] |
| 6 | ['FLT1 -> STAT1->AAK1 -'] |
| 7 | ['FLT1 -> STAT1->AAK1 -'] |
| 8 | ['EGFR -> EGR1->NR1H3 -> STAT1 -> NDUFC1 -'] |
| 9 | ['EGFR -> EGR1->NR1H3 -> STAT1 -> NDUFC1 -'] |
| 10 | ['FLT1 -> STAT1->DENND1B -'] |
| 11 | ['FLT1 -> STAT1->DENND1B -'] |
| 12 | ['FLT1 -> STAT1->ATF3 -'] |
| 13 | ['FLT1 -> STAT1->BRCA1 -'] |
| 14 | ['FLT1 -> STAT1->OASL -'] |
| 15 | ['FLT1 -> STAT1->SECTM1 -'] |
| 16 | ['FLT1 -> STAT1->MAGT1 -'] |
| 17 | ['FLT1 -> STAT1->MAGT1 -'] |
| 18 | ['FLT1 -> STAT1->BRCA1 -'] |
| 19 | ['FLT1 -> STAT1->IFITM1 -'] |
| 20 | ['FLT1 -> STAT1->IFITM1 -'] |
| 21 | ['FLT1 -> STAT1->OASL -'] |
| 22 | ['FLT1 -> STAT1->SECTM1 -'] |
| 23 | ['FLT1 -> STAT1->NR1D1 -'] |
| 24 | ['FLT1 -> STAT1->NR1D1 -'] |
| 25 | ['FLT1 -> STAT1->ARHGAP10 -'] |
| 26 | ['FLT1 -> STAT1->ARHGAP10 -'] |
| 27 | ['F11R -> PRKCA->SYK -'] |
| 28 | ['FLT1 -> STAT1->ATF3 -> MIR22HG -'] |
| 29 | ['FLT1 -> STAT1->ATF3 -> MIR22HG -'] |
| 30 | ['IGF1R -> STAT3->EGR1 -> NR1H3 -> STAT1 -> NDUFC1 -'] |
| 31 | ['IGF1R -> STAT3->EGR1 -> NR1H3 -> STAT1 -> NDUFC1 -'] |
| 32 | ['EPHB1 -> STAT3->EGR1 -> NR1H3 -> STAT1 -> NDUFC1 -'] |
| 33 | ['EPHB1 -> STAT3->EGR1 -> NR1H3 -> STAT1 -> NDUFC1 -'] |
| 34 | ['IGF1R -> STAT3->CHCHD10 -'] |
| 35 | ['EPHB1 -> STAT3->CHCHD10 -'] |
| 36 | ['EPHB1 -> STAT3->CHCHD10 -'] |
| 37 | ['IGF1R -> STAT3->CHCHD10 -'] |
| 38 | ['EGFR -> EGR1->NR1H3 -> STAT1 -> USP12 -'] |
| 39 | ['EGFR -> EGR1->NR1H3 -> STAT1 -> USP12 -'] |
| 40 | ['FLT1 -> STAT1->ATF3 -> HAUS2 -'] |
| 41 | ['FLT1 -> STAT1->ATF3 -> DOCK11 -'] |
| 42 | ['FLT1 -> STAT1->ATF3 -> HAUS2 -'] |
| 43 | ['FLT1 -> STAT1->ATF3 -> DOCK11 -'] |
| 44 | ['FLT1 -> STAT1->ATF3 -> PPP1R15A -'] |
| 45 | ['FLT1 -> STAT1->ATF3 -> SIK1 -'] |
| 46 | ['FLT1 -> STAT1->ATF3 -> SLC13A3 -'] |
| 47 | ['FLT1 -> STAT1->ATF3 -> TWIST1 -'] |
| 48 | ['FLT1 -> STAT1->ATF3 -> SLC13A3 -'] |
| 49 | ['FLT1 -> STAT1->ATF3 -> TAGLN2 -'] |
| 50 | ['FLT1 -> STAT1->BRCA1 -> SMARCA2 -'] |
| 51 | ['FLT1 -> STAT1->ATF3 -> PPP1R15A -'] |
| 52 | ['FLT1 -> STAT1->ATF3 -> TWIST1 -'] |
| 53 | ['FLT1 -> STAT1->ATF3 -> SIK1 -'] |
| 54 | ['EGFR -> EGR1->NR1H3 -> STAT1 -> ATF3 -> MAPK10 -'] |
| 55 | ['FLT1 -> STAT1->IFITM1 -> CR2 -'] |
| 56 | ['FLT1 -> STAT1->ATF3 -> TAGLN2 -'] |
| 57 | ['EGFR -> EGR1->NR1H3 -> STAT1 -> ATF3 -> MAPK10 -'] |
| 58 | ['RYR1 -> CAMK2G->FOS -> TRNP1 -'] |
| 59 | ['ESR2 -> FGR'] |
| 60 | ['FLT1 -> STAT1->NR1D1 -> PCDH19 -'] |
| 61 | ['RYR1 -> CAMK2G->FOS -> TRNP1 -'] |
| 62 | ['FLT1 -> STAT1->NR1D1 -> PCDH19 -'] |
| 63 | ['EGFR -> CCR5->HPRT1 -'] |
| 64 | ['NPBWR1 -> CCR5->HPRT1 -'] |
| 65 | ['EGFR -> EGR1->NR1H3 -> STAT1 -'] |
| 66 | ['LEPR -> IGF1R->STAT3 -> EGR1 -> NR1H3 -> STAT1 -> NDUFC1 -'] |
| 67 | ['LEPR -> IGF1R->STAT3 -> EGR1 -> NR1H3 -> STAT1 -> NDUFC1 -'] |
| 68 | ['CXCR6 -> CCR5->HPRT1 -'] |
| 69 | ['RYR1 -> CAMK2G->FOS -> ZFP36L2 -'] |
| 70 | ['RYR1 -> CAMK2G->FOS -> ZFP36L2 -'] |
| 71 | ['TNFRSF11B -> PAX6->GNG5 -'] |
| 72 | ['HCRTR1 -> NPFFR1->GNG5 -'] |
| 73 | ['TNFRSF11B -> PAX6->GNG5 -'] |
| 74 | ['EPHB1 -> STAT3->EGR1 -> NR1H3 -> STAT1 -> USP12 -'] |
| 75 | ['EPHB1 -> STAT3->EGR1 -> NR1H3 -> STAT1 -> USP12 -'] |
| 76 | ['IGF1R -> STAT3->EGR1 -> NR1H3 -> STAT1 -> USP12 -'] |
| 77 | ['IGF1R -> STAT3->EGR1 -> NR1H3 -> STAT1 -> USP12 -'] |
| 78 | ['KIT -> BLNK->CAMK2A -'] |
| 79 | ['MET -> CXCR6->CCR5 -'] |
| 80 | ['IGF1R -> STAT3->EGR1 -> NR1H3 -> STAT1 -> ATF3 -> MAPK10 -'] |
| 81 | ['EPHB1 -> STAT3->EGR1 -> NR1H3 -> STAT1 -> ATF3 -> MAPK10 -'] |
| 82 | ['IGF1R -> STAT3->EGR1 -> NR1H3 -> STAT1 -> ATF3 -> MAPK10 -'] |
| 83 | ['EPHB1 -> STAT3->EGR1 -> NR1H3 -> STAT1 -> ATF3 -> MAPK10 -'] |
| 84 | ['EGFR -> EGR1->NR1H3 -> STAT1 -> AAK1 -'] |
| 85 | ['EGFR -> EGR1->NR1H3 -> STAT1 -> AAK1 -'] |
| 86 | ['PTK2B -> ASAP1->REPS2 -'] |
| 87 | ['FLT1 -> STAT1->BRCA1 -> SMARCA2 -> DPF3 -'] |
| 88 | ['LEPR -> IGF1R->STAT3 -> CHCHD10 -'] |
| 89 | ['AR -> KLF6'] |
| 90 | ['EGFR -> EGR1->NR1H3 -> STAT1 -> OASL -'] |
| 91 | ['EGFR -> EGR1->NR1H3 -> STAT1 -> MAGT1 -'] |
| 92 | ['RYR1 -> CAMK2G->FOS -> LY9 -'] |
| 93 | ['LEPR -> IGF1R->STAT3 -> CHCHD10 -'] |
| 94 | ['EGFR -> EGR1->NR1H3 -> STAT1 -> DENND1B -'] |
| 95 | ['EGFR -> EGR1->NR1H3 -> STAT1 -> NR1D1 -'] |
| 96 | ['EGFR -> EGR1->NR1H3 -> STAT1 -> IFITM1 -'] |
| 97 | ['EGFR -> EGR1->NR1H3 -> STAT1 -> SECTM1 -'] |
| 98 | ['EGFR -> EGR1->NR1H3 -> STAT1 -> IFITM1 -'] |
| 99 | ['EGFR -> EGR1->NR1H3 -> STAT1 -> DENND1B -'] |
| 100 | ['EGFR -> EGR1->NR1H3 -> STAT1 -> ATF3 -'] |

**Supplementary Table 5: The top 100 paths discovered by the PathFinder on TAFE4_ast.**

| **Order** | **Path** |
| --- | --- |
| 0 | ['NLGN1 -> COL4A5'] |
| 1 | ['FLT1 -> STAT1->NDUFC1 -'] |
| 2 | ['FLT1 -> STAT1->NDUFC1 -'] |
| 3 | ['AR -> VPS37B'] |
| 4 | ['EPHB1 -> STAT3->FOXO1 -> CEBPB -> SMC2 -> SOX2 -> DIP2A -'] |
| 5 | ['IGF1R -> STAT3->CHCHD10 -'] |
| 6 | ['FLT1 -> STAT1->NR1D1 -> PCDH19 -'] |
| 7 | ['IGF1R -> STAT3->FOXO1 -> CEBPB -> SMC2 -> SOX2 -> DIP2A -'] |
| 8 | ['EPHB1 -> STAT3->CHCHD10 -'] |
| 9 | ['IGF1R -> STAT3->CHCHD10 -'] |
| 10 | ['EPHB1 -> STAT3->FOXO1 -> CEBPB -> SMC2 -> SOX2 -> DIP2A -'] |
| 11 | ['EPHB1 -> STAT3->CHCHD10 -'] |
| 12 | ['EPHB1 -> STAT3->EGR1 -> NR1H3 -> STAT1 -> NDUFC1 -'] |
| 13 | ['FLT1 -> STAT1->NR1D1 -> PCDH19 -'] |
| 14 | ['EPHB1 -> STAT3->PCDH7 -'] |
| 15 | ['IGF1R -> STAT3->EGR1 -> NR1H3 -> STAT1 -> NDUFC1 -'] |
| 16 | ['IGF1R -> STAT3->FOXO1 -> CEBPB -> SMC2 -> SOX2 -> DIP2A -'] |
| 17 | ['FLT1 -> STAT1->NR1D1 -'] |
| 18 | ['IGF1R -> STAT3->EGR1 -> NR1H3 -> STAT1 -> NDUFC1 -'] |
| 19 | ['IGF1R -> STAT3->PCDH7 -'] |
| 20 | ['IGF1R -> STAT3->EGR1 -> NR1H3 -> STAT1 -> NR1D1 -> PCDH19 -'] |
| 21 | ['EPHB1 -> STAT3->EGR1 -> NR1H3 -> STAT1 -> NR1D1 -> PCDH19 -'] |
| 22 | ['EPHB1 -> STAT3->EGR1 -> EMP3 -'] |
| 23 | ['EPHB1 -> STAT3->EGR1 -> NR1H3 -> STAT1 -> NDUFC1 -'] |
| 24 | ['IGF1R -> STAT3->EGR1 -> CYP51A1 -'] |
| 25 | ['LEPR -> IGF1R->STAT3 -> FOXO1 -> CEBPB -> SMC2 -> SOX2 -> DIP2A -'] |
| 26 | ['TGFBR1 -> SPEM1'] |
| 27 | ['IGF1R -> STAT3->EGR1 -> CYP51A1 -'] |
| 28 | ['EPHB1 -> STAT3->EGR1 -'] |
| 29 | ['FLT1 -> STAT1->NR1D1 -'] |
| 30 | ['EPHB1 -> STAT3->FOXO1 -> CEBPB -> SMC2 -> SOX2 -> RNF43 -'] |
| 31 | ['EPHB1 -> STAT3->EGR1 -> NR1H3 -> STAT1 -'] |
| 32 | ['IGF1R -> STAT3->EGR1 -> CRIP2 -'] |
| 33 | ['EPHB1 -> STAT3->FOXO1 -> CEBPB -> MTSS1L -'] |
| 34 | ['EPHB1 -> STAT3->EGR1 -> HIVEP1 -'] |
| 35 | ['IGF1R -> STAT3->FOXO1 -> CEBPB -> SMC2 -> SOX2 -'] |
| 36 | ['EPHB1 -> STAT3->FOXO1 -> CEBPB -> PLEKHG2 -'] |
| 37 | ['EPHB1 -> STAT3->FOXO1 -> CEBPB -> SMC2 -> SOX2 -> NIP7 -'] |
| 38 | ['IGF1R -> STAT3->FOXO1 -> CEBPB -> SMC2 -> SOX2 -> TRIO -'] |
| 39 | ['IGF1R -> STAT3->FOXO1 -> CEBPB -> ACADVL -'] |
| 40 | ['IGF1R -> STAT3->FOXO1 -> CEBPB -> SMC2 -> SOX2 -> TRIO -'] |
| 41 | ['AR -> NRXN3'] |
| 42 | ['IGF1R -> STAT3->EGR1 -> EMP3 -'] |
| 43 | ['EPHB1 -> STAT3->EGR1 -> ATP6V1F -'] |
| 44 | ['IGF1R -> STAT3->EGR1 -> NR1H3 -> STAT1 -'] |
| 45 | ['EPHB1 -> STAT3->BTBD9 -'] |
| 46 | ['EPHB1 -> STAT3->FOXO1 -> CEBPB -> MTSS1L -'] |
| 47 | ['IGF1R -> STAT3->FOXO1 -> CEBPB -> PLEKHG2 -'] |
| 48 | ['IGF1R -> STAT3->EGR1 -> ICAM5 -'] |
| 49 | ['IGF1R -> STAT3->EGR1 -> EMP3 -'] |
| 50 | ['LEPR -> IGF1R->STAT3 -> CHCHD10 -'] |
| 51 | ['EGFR -> EGR1->NR1H3 -> STAT1 -> NDUFC1 -'] |
| 52 | ['EPHB1 -> STAT3->PCDH7 -'] |
| 53 | ['LEPR -> IGF1R->STAT3 -> FOXO1 -> CEBPB -> SMC2 -> SOX2 -> DIP2A -'] |
| 54 | ['EPHB1 -> STAT3->EGR1 -> CRB2 -'] |
| 55 | ['IGF1R -> STAT3->EGR1 -> NR1H3 -> STAT1 -> NR1D1 -'] |
| 56 | ['IGF1R -> STAT3->RAPGEFL1 -'] |
| 57 | ['KIT -> BLNK->CAMK2A -'] |
| 58 | ['EPHB1 -> STAT3->EGR1 -> CYP51A1 -'] |
| 59 | ['EPHB1 -> STAT3->EGR1 -> HIVEP1 -> DUSP10 -'] |
| 60 | ['FLT1 -> STAT1->MAGT1 -'] |
| 61 | ['EPHB1 -> STAT3->EGR1 -> HIVEP1 -'] |
| 62 | ['IGF1R -> STAT3->EGR1 -> SPOCK1 -'] |
| 63 | ['IGF1R -> STAT3->EGR1 -> NR1H3 -> STAT1 -> ARHGAP10 -'] |
| 64 | ['EPHB1 -> STAT3->EGR1 -> NR1H3 -> STAT1 -> NR1D1 -> PCDH19 -'] |
| 65 | ['LEPR -> IGF1R->STAT3 -> EGR1 -'] |
| 66 | ['EPHB1 -> STAT3->FOXO1 -> CEBPB -> SMC2 -> SOX2 -> IQSEC3 -'] |
| 67 | ['FLT1 -> STAT1->BRCA1 -> SMARCA2 -'] |
| 68 | ['LEPR -> IGF1R->STAT3 -> EGR1 -> HIVEP1 -'] |
| 69 | ['EPHB1 -> STAT3->FOXO1 -> CEBPB -> TMEM245 -'] |
| 70 | ['EPHB1 -> STAT3->MET -> CXCR6 -> CCR5 -> HPRT1 -'] |
| 71 | ['IGF1R -> STAT3->FOXO1 -> CEBPB -> PLEKHG2 -'] |
| 72 | ['EPHB1 -> STAT3->STEAP4 -> ATP7B -> CLU -'] |
| 73 | ['LEPR -> IGF1R->STAT3 -> FOXO1 -> CEBPB -> SMC2 -> SOX2 -> IFITM2 -'] |
| 74 | ['LEPR -> IGF1R->STAT3 -> EGR1 -> NR1H3 -> STAT1 -> ATF3 -> MAPK10 -'] |
| 75 | ['FLT1 -> STAT1->ATF3 -> SIK1 -'] |
| 76 | ['IGF1R -> STAT3->EGR1 -> HIVEP1 -> DUSP10 -'] |
| 77 | ['IGF1R -> STAT3->EGR1 -> EXPH5 -'] |
| 78 | ['EPHB1 -> STAT3->FOXO1 -> CEBPB -> SMC2 -> SOX2 -> SP9 -'] |
| 79 | ['IGF1R -> STAT3->EGR1 -'] |
| 80 | ['EPHB1 -> STAT3->FOXO1 -> CEBPB -> SMC2 -'] |
| 81 | ['IGF1R -> STAT3->EGR1 -'] |
| 82 | ['IGF1R -> STAT3->ETV6 -'] |
| 83 | ['IGF1R -> STAT3->EGR1 -> NR1H3 -> STAT1 -> DENND1B -'] |
| 84 | ['EPHB1 -> STAT3->EGR1 -> EMP3 -'] |
| 85 | ['IGF1R -> STAT3->EGR1 -> NR1H3 -> STAT1 -> OASL -'] |
| 86 | ['LEPR -> IGF1R->STAT3 -> BTBD9 -'] |
| 87 | ['IGF1R -> STAT3->EGR1 -> NR1H3 -> STAT1 -> ATF3 -> MIR22HG -'] |
| 88 | ['EPHB1 -> STAT3->EGR1 -> NR1H3 -> STAT1 -> DENND1B -'] |
| 89 | ['LEPR -> IGF1R->STAT3 -> EGR1 -> NR1H3 -> STAT1 -> MAGT1 -'] |
| 90 | ['LEPR -> IGF1R->STAT3 -> FGF19 -'] |
| 91 | ['LEPR -> IGF1R->STAT3 -> EGR1 -> NR1H3 -> STAT1 -> NDUFC1 -'] |
| 92 | ['EPHB1 -> STAT3->EGR1 -> CRIP2 -'] |
| 93 | ['EPHB1 -> STAT3->EGR1 -> SPOCK1 -'] |
| 94 | ['ERBB3 -> PPP2R2B'] |
| 95 | ['LEPR -> IGF1R->STAT3 -> CHCHD10 -'] |
| 96 | ['EPHB1 -> STAT3->EGR1 -> ATP6V1F -'] |
| 97 | ['LEPR -> IGF1R->STAT3 -> EGR1 -> NR1H3 -> STAT1 -> NR1D1 -'] |
| 98 | ['LEPR -> IGF1R->STAT3 -> EGR1 -> EMP3 -'] |
| 99 | ['EGFR -> EGR1->NR1H3 -> STAT1 -> NDUFC1 -'] |
| 100 | ['LEPR -> IGF1R->STAT3 -> STEAP4 -> ATP7B -> CLU -'] |
